# MIFAD-Net: Multi-Layer Interactive Feature Fusion Network With Angular Distance Loss for Face Emotion Recognition

**DOI:** 10.3389/fpsyg.2021.762795

**Published:** 2021-10-22

**Authors:** Weiwei Cai, Ming Gao, Runmin Liu, Jie Mao

**Affiliations:** ^1^College of Sports Engineering and Information Technology, Wuhan Sports University, Wuhan, China; ^2^School of Logistics and Transportation, Central South University of Forestry and Technology, Changsha, China; ^3^College of Sports Science and Technology, Wuhan Sports University, Wuhan, China

**Keywords:** face emotion, emotion recognition, multi-layer interactive, feature fusion, deep learning, neural networks

## Abstract

Understanding human emotions and psychology is a critical step toward realizing artificial intelligence, and correct recognition of facial expressions is essential for judging emotions. However, the differences caused by changes in facial expression are very subtle, and different expression features are less distinguishable, making it difficult for computers to recognize human facial emotions accurately. Therefore, this paper proposes a novel multi-layer interactive feature fusion network model with angular distance loss. To begin, a multi-layer and multi-scale module is designed to extract global and local features of facial emotions in order to capture part of the feature relationships between different scales, thereby improving the model's ability to discriminate subtle features of facial emotions. Second, a hierarchical interactive feature fusion module is designed to address the issue of loss of useful feature information caused by layer-by-layer convolution and pooling of convolutional neural networks. In addition, the attention mechanism is also used between convolutional layers at different levels. Improve the neural network's discriminative ability by increasing the saliency of information about different features on the layers and suppressing irrelevant information. Finally, we use the angular distance loss function to improve the proposed model's inter-class feature separation and intra-class feature clustering capabilities, addressing the issues of large intra-class differences and high inter-class similarity in facial emotion recognition. We conducted comparison and ablation experiments on the FER2013 dataset. The results illustrate that the performance of the proposed MIFAD-Net is 1.02–4.53% better than the compared methods, and it has strong competitiveness.

## Introduction

Emotions are extremely important in everyday life. It is often necessary to accompany the correct understanding of other people's emotions in the process of human daily communication and behavior judgment, and facial expressions contain a lot of information about emotions and mental states. Therefore, it is possible to say that recognizing facial expressions (Crivelli et al., 2017; Chengeta and Viriri, 2019; González-Lozoya et al., 2020) is the key to understanding emotions. According to psychologists' research, only 7% of information in the process of human communication comes from pure language expression, 38% from sound information such as speech pitch, and 55% from visuals such as facial emotions. The content has been communicated. As a result, accurate recognition of facial expressions is critical for understanding information in human communication.

Facial emotion recognition (Sreedharan et al., 2018; Jain et al., 2019) can be used in a variety of situations. In terms of human-computer interaction, accurate facial expression recognition to determine human emotions can make machines more appropriate, accurate, and effective in interacting with humans, resulting in a more natural interaction. Interaction and exchange with humans. In terms of security scenarios, it is possible to effectively identify suspects with criminal intent in public by accurately identifying facial expressions and subtle expressions. In terms of transportation, it is possible to better judge whether a driver is fatigued by recognizing the facial expressions of drivers of vehicles such as vehicles (Theagarajan et al., 2017; Zepf et al., 2020). Furthermore, facial expression recognition has gotten a lot of attention in the advertising (Hamelin et al., 2017) and marketing, automation, and communications fields.

In recent years, facial emotion recognition based on deep learning technology (Cai and Wei, 2020; Cai et al., 2021; Zhang et al., 2021) has made great progress, but there are still many problems to be solved. For example, the recognition accuracy in real scenes is still not ideal. Among the basic emotion categories of human faces, negative emotions, including angry, disgust, disappointment, etc., have no relatively uniform standard for facial expressions, and feature differences are minimal, which are not conducive to computer feature learning and are often difficult to correctly recognize. Furthermore, because the face area occupies a relatively small area in an image, the data used for facial emotion recognition model training has a small input size. The current convolutional neural network (CNN) model (Bendjoudi et al., 2020; Kollias and Zafeiriou, 2020; Kwon, 2021) necessitates a relatively large image size as input. Excessive use of interpolation and other methods to increase image size results in more calculations. On the contrary, the recognition effect has not improved significantly.

Based on the above observations, in this paper, a multi-layer and multi-scale module is designed to extract the global and local features of facial expressions to capture part of the feature relationships between different scales, thereby enhancing the model's ability to discriminate subtle features of facial expressions. Secondly, in view of the problem of loss of useful feature information due to layer-by-layer convolution and pooling of CNNs, a hierarchical interactive feature fusion module is designed. The attention mechanism (Gao et al., 2021; Liu et al., 2021) is used between convolutional layers at different levels to control the network. Strengthen the saliency information of different characteristics in the Internet and suppress irrelevant information, thereby improving the discriminative ability of the network. Finally, for the problem of large intra-class differences and high inter-class similarity in facial expression recognition, we use the angular distance loss function to improve the capabilities of the proposed algorithm for feature separation between classes and clustering of features within classes.

The main innovations of this paper are as follows:

(1) To address the problem of subtle differences in facial emotion causing difficulty in classification, we designed a multi-layer and multi-scale module to extract global and local facial emotion features to capture partial feature relationships between different scales, thereby improving the model's ability to discriminate subtle facial emotion features.
(2) To address the issue of loss of useful feature information caused by layer-by-layer convolution and pooling of convolutional neural networks, we created a hierarchical interactive feature fusion module that controls the network using the attention mechanism between convolutional layers at different levels. The importance of various characteristics is increased, while irrelevant information is reduced.
(3) We use the angular distance loss function to improve the capabilities of the proposed algorithm for feature separation between classes and clustering of features within classes, with the goal of addressing the problem of large intra-class differences and high inter-class similarity in face emotion recognition.

The remainder of this article is organized as follows. We introduce relevant work in section Related work, describe the proposed algorithm in section Methodology, and present the experimental results in section Experiments and Results. This paper's research conclusions are presented in section Conclusion.

## Related work

### Emotion Recognition Based on Traditional Machine Learning

The emotion recognition method based on traditional machine learning (Bota et al., 2019; Kerkeni et al., 2019; Domínguez-Jiménez et al., 2020) is mainly to manually extract the emotion image features and then use the appropriate classification algorithm to classify the emotion. The specific method is to manually select some appropriate feature extraction operators to extract facial features, and then do appropriate dimension reduction processing on the extracted facial features, and finally select a classifier to classify the facial features after dimension reduction. Kumar et al. (2016) believed that features in different regions of the face contribute to expression recognition to different extent, and important locations have important feature information, such as mouth and eyes. Therefore, they proposed a weighted projection LBP feature extraction algorithm for different information regions, and improved the accuracy of expression recognition by cascading the weighted features of different regions. As a linear filter, Gabor is robust to light changes, and it can also change the frequency and direction to analyze texture features. Zhang et al. (2014) proposed an emotion recognition method in the case of occlusion. This method adopted Monte Carlo algorithm to extract features based on Gabor template in the image, and the features obtained were robust for occlusion. Harit et al. (2018) proposed an automatic expression recognition algorithm that constructed an expression feature space using multiple Gabor filters at reference points before sending it to a neural network for classification. Wang et al. (2017) developed a multi-scale geometric feature extraction method, which mapped the original expression information to geometric feature functions, and then used the feature functions for further analysis. Tarannum et al. (2016) took the Euclidean distance between various facial regions as a feature and then used Canberra distance to classify the features.

After the expression feature extraction is completed, the classifier can specifically classify the expression feature into a certain expression category. Common facial expression classifiers include SVM algorithm (Xu et al., 2012), KNN algorithm and so on. Liew and Yairi (2015) proposed to use SVM as a classifier to classify the Hog expression features extracted in the early stage. This method has achieved good results on the JAFFE data set. Ouellet (2014) replaced the sofhnax classification layer of the Alexnet network with SVM Multi-classifiers, and achieved better recognition results on the CK+ expression library. Rieger et al. (2014) used a pattern recognition paradigm with spectral feature extraction and a set of KNN classifiers to investigate speech-based emotion recognition, and found that using two KNNs yielded the best results.

### Emotion Recognition Based on Deep Learning

CNNs, in contrast to traditional machine learning methods, can automatically extract deep-level features of facial expressions by constructing multiple convolutional layers. On the one hand, it avoids errors caused by artificial feature extraction, on the other hand, it has strong robustness and generalization ability, so it has gradually become the mainstream method. Researchers are beginning to study applying deep learning to facial expression recognition tasks. Mollahosseini et al. (2016) proposed a 7-layer convolutional neural network that combined AlexNet and GoogleNet models and then verified them using seven public expression data sets, which was faster and more accurate than a traditional convolutional neural network. Zhang et al. (2019) used a stacked hybrid autoencoder to recognize expression. Three encoders were used in the network structure: a denoising autoencoder, a sparse autoencoder, and an autoencoder. The feature extraction was done with the denoising encoder, and the sparse autoencoder was utilized for cascading to extract more abstract sparse features. Tang (2013) combined CNNs and svm, and used the hinge loss instead of the common cross-entropy loss function in convolutional neural networks. On the FER2013 data set, the detection rate was 71.2 percent, and the team won the Kaggle facial expression recognition competition in 2013. After analyzing the network structure of expression recognition based on deep CNNs, Pramerdorfer and Kampel (2016) improved the classic ResNet and input a single face image to extract facial expression features, achieving an average recognition rate of 72.4% on the FER2013 data set. Lee et al. (2019) proposed a deep network for context-aware emotion recognition, which not only uses human facial expressions, but also uses context information in a joint and enhanced manner, which effectively improves the performance of emotion recognition.

Although deep learning technology has achieved excellent results in face emotion recognition tasks, the differences caused by facial expression changes are very subtle, and different facial expression features are not distinguishable, resulting in low face emotion recognition accuracy.

## Methodology

**Figure 1:**
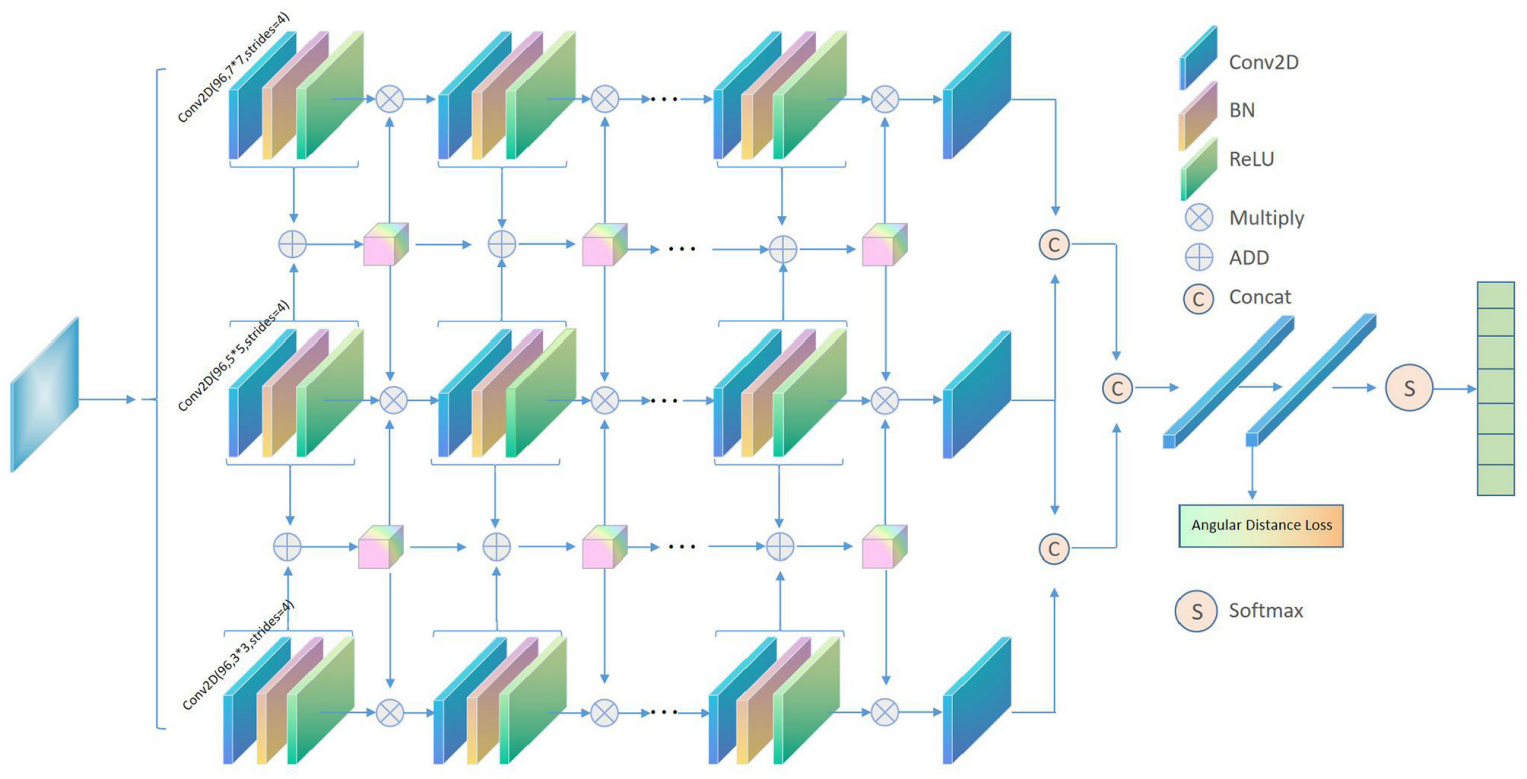
Schematic diagram of the proposed MIFAD-Net algorithm.

The MIFAD-Net model of this article is shown in Figure 1. The three columns show that networks with different thickness scales using convolutional scale kernels of 7, 5, and 3 can extract more refined facial emotion features. Each column of the network has six convolutional layers and five BN layers. The three-column network has a common facial emotion image input. After that, the feature maps of the last three convolutional layers of the same depth position of the three-column network are interacted through the feature splicing strategy to integrate different cross-layer features of the same network and different networks to capture the deep connection between different levels to facilitate subsequent facial emotions Feature classification. In addition, the three-column network also interacts with the collection of features through the addition strategy, and uses the attention mechanism to focus on the effective features. Finally, we are employing the angular distance loss function to improve the model's capacity to segregate features between classes and cluster features within classes, which is a challenge with big intra-class variances in facial expression recognition and high similarity between classes. Then, using Softmax, establish the face expression category. After that, we'll go over the proposed model in more detail.

### Multi-Scale and Multi-Layer Interactive Feature Fusion

**Figure 2:**
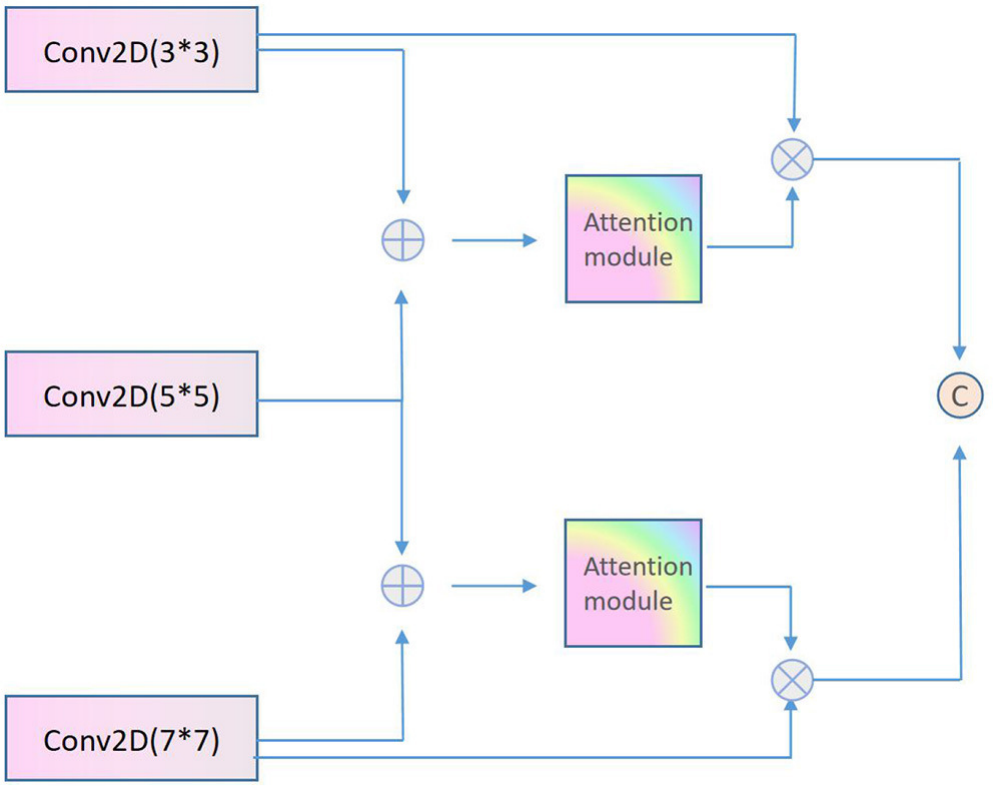
Schematic diagram of multi-scale and multi-layer interactive feature fusion.

This paper proposes a multi-scale and multi-layer interactive feature fusion module, as shown in Figure 2, to make better use of the different scale features of facial emotion images. The module first merges the feature maps 3^*^3, 5^*^5, and 7^*^7 of the three coarse and fine scale networks through 3 × 3. One branch is activated by Sigmoid to generate feature weights and then multiplied by the three feature map elements to obtain the re-calibrated features. Figure, and finally get the final output through the feature splicing strategy. The module can self-update learning according to back propagation, and automatically select the multi-scale features that each branch needs to be fused.

#### Multi-Scale Convolution

**Figure 3:**
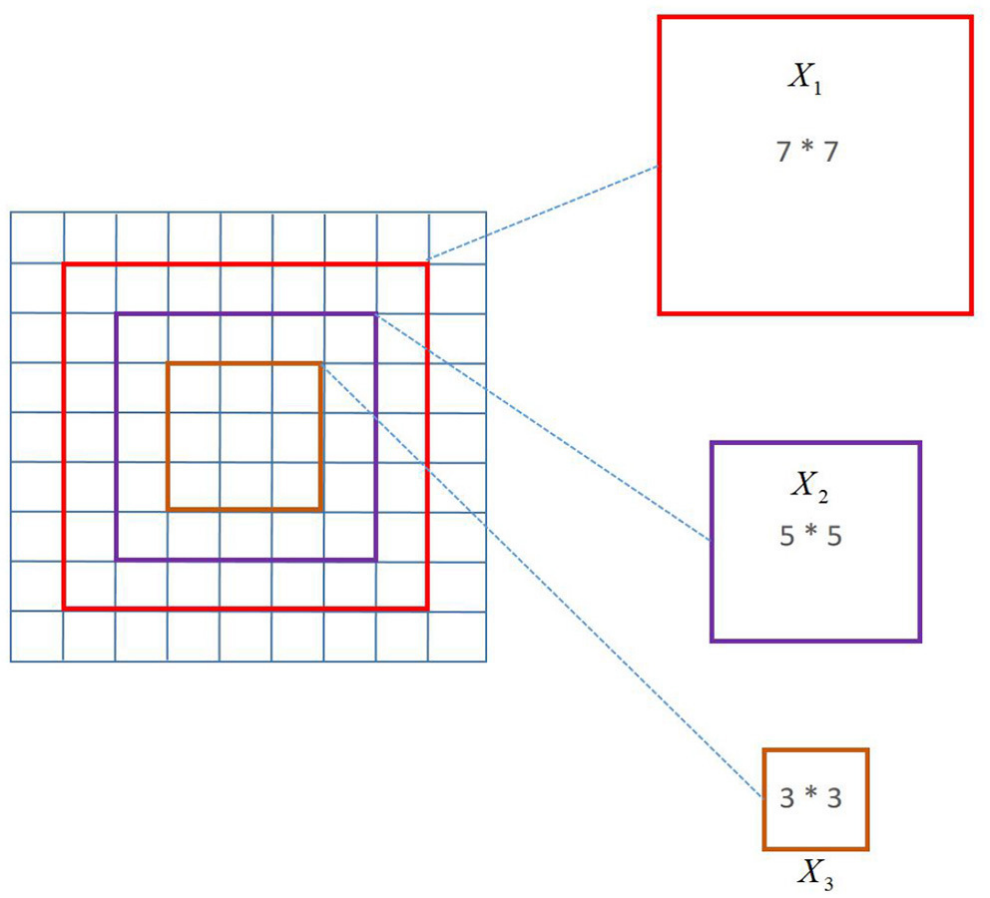
Schematic diagram of multi-scale convolution.

The use of a multi-scale convolution kernel has two major advantages, as discussed in this article. First and foremost, the multi-scale convolution kernel has the advantage of allowing different-sized convolution kernels to extract multiple scales of facial emotion picture data, allowing the filter to extract and learn richer high-dimensional features. Second, the convolutional neural network trains the model by learning the filter's parameters (weight and offset), i.e., continuously learning the filter's parameters to acquire the ideal value closest to the label. This article employs a multi-scale convolution kernel with the goal of allowing a single convolution layer to have several filters, so diversifying the weight and bias learning, and thereby extracting and learning the semantic aspects of facial emotion photos fully and efficiently. A schematic diagram of multi-scale convolution is shown in Figure 3.

To achieve the best results, multi-scale inference approaches are commonly used in computer vision models. Fine details are better predicted at larger sizes, larger objects are better predicted at smaller sizes, and the network's receiving field can interpret the scene better at smaller sizes. The 3^*^3, 5^*^5, and 7^*^7 scale convolution kernels were employed in this article's multi-scale convolution. The following is the calculating formula:


(1) $$\begin{array}{l} Y^{H\times W}=\phi \left\{\overset{n}{\underset{i=0}{\sum }}w_{ij}^{H\times W}*x_{i}^{H\times W}+b_{\text{j}}\right\} \end{array}$$

where *H* × *W* represents the size of the convolution kernel.

#### Attention Mechanism

**Figure 4:**
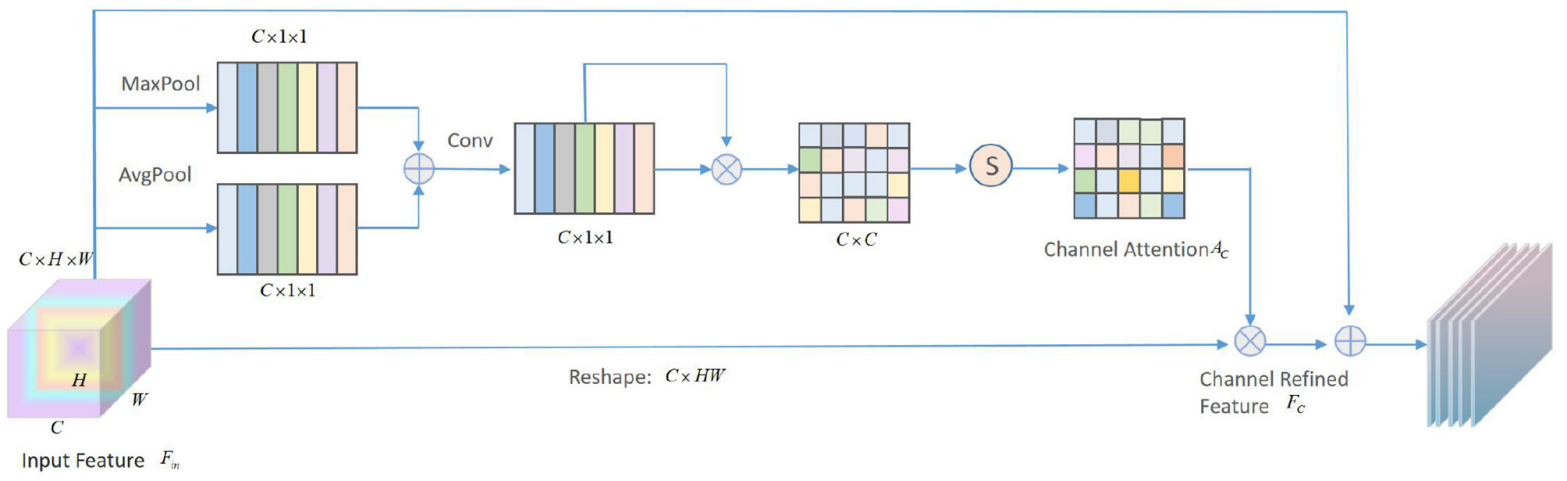
Schematic diagram of Channel attention module.

Different areas in facial emotion images have different weights for different tasks. The higher the relevance to the task, the more important the field is. In this article, the attention module we designed is composed of cascaded channel attention and spatial attention. The schematic diagram of the channel attention mechanism is shown in Figure 4.

The given input feature $F_{in}\in R^{C\times H\times W}$ is fed into the proposed channel attention module. First, global average pooling (GAP) and max-pooling are used to compress the feature map along the spatial axis in parallel to generate two *C* × 1 × 1 dimensional feature vectors $F_{a}^{c}$ And $F_{m}^{c}$, and then perform element-wise summation to obtain the aggregate characteristic $F_{s}^{c}$ of all features. After that, go through a convolutional layer with a kernel size of 1 × 1, and then execute PReLU and BatchNorm to get the middle feature map $F_{p}^{c}$, then:


(2) $$\begin{array}{l} F_{p}^{c}=C\text{onv}\left(F_{a}^{c}⊕F_{m}^{c}\right)=\phi^{1\times 1}\left(F_{a}^{c}⊕F_{m}^{c}\right) \end{array}$$

where ⊕ represents the element summation, and ϕ represents the convolution operation. Then, the $F_{p}^{c}$ is deformed and transposed to obtain two feature maps with dimensions *C*×1 and 1×*C*, and then matrix multiplication and softmax operations are performed to obtain the channel attention matrix *A*_*c*_. The calculation equation is as follows:


(3) $$\begin{array}{l} A_{c}=soft\;\text{max}(F_{p}^{c}⊗{\left(F_{p}^{c}\right)}^{T}) \end{array}$$

where ⊗ represents matrix multiplication, and the following equation can be obtained:


(4) $$\begin{array}{l} A_{{\text{c}}_{\text{i},\text{j}}}=\frac{\text{exp}\left({\left(F_{p}^{c}\right)}_{i}{\left(F_{p}^{c}\right)}_{j}^{T}\right)}{\sum_{\text{i}=1}^{C}\text{exp}\left({\left(F_{p}^{c}\right)}_{i}{\left(F_{p}^{c}\right)}_{j}^{T}\right)} \end{array}$$

where *A*_*c*_*i,j*__ represents the influence of the *i*-th channel on the *j*-th channel. Finally, the input feature *F*_*in*_ is multiplied by the channel attention matrix *A*_*c*_, and then the refined feature $F_{C}\in R^{C\times H\times W}$ of the channel is obtained through learning.


(5) $$\begin{array}{l} F_{C}=F_{in}⊕\left(\alpha \left(A_{c}⊗F_{in}\right)\right) \end{array}$$

where α is a learnable parameter, and generally the initial value is set to 0 to reduce the difficulty of the convergence process of the first few training cycles. In this way, the channel attention matrix *A*_*c*_ can be regarded as a kernel selector to select the filter used to describe the emotional characteristics of the face.

**Figure 5:**
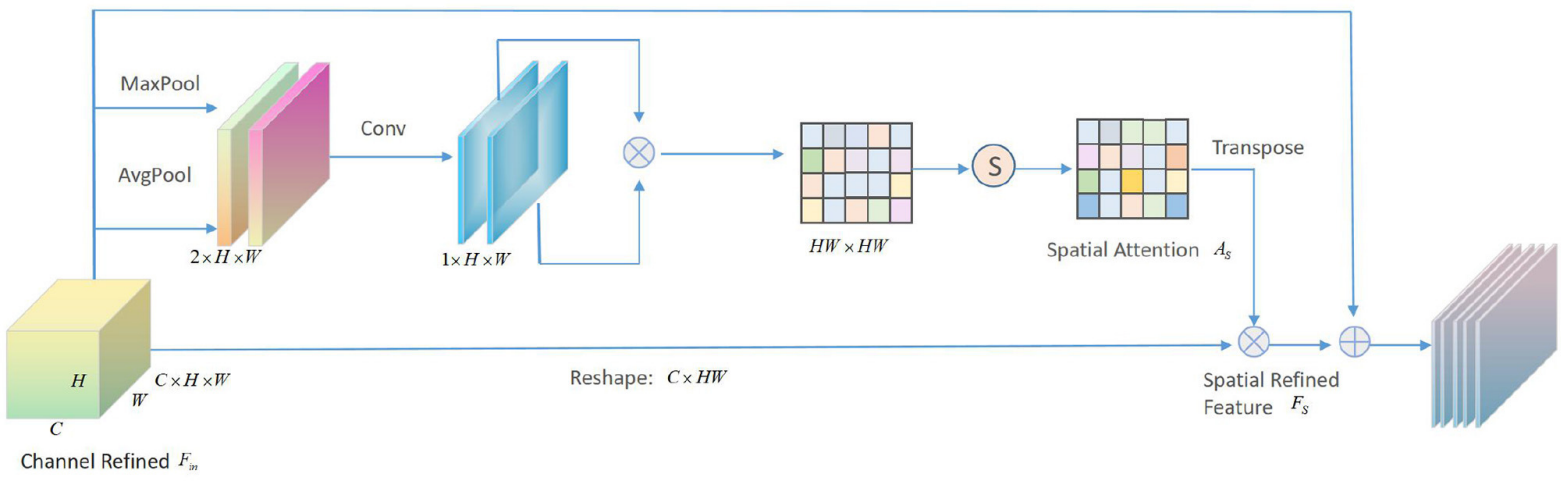
Schematic diagram of spatial attention module.

In addition to channel attention, we also cascade a spatial attention module to learn the relationship between the spatial structure of the intermediate feature maps. The spatial attention module, which can be used in conjunction with the channel attention module, generates a spatial attention matrix to focus attention on the part that best represents facial feature information. Apply average pooling and maximum pooling along the channel axis, cascade them to get an effective feature descriptor, and then use matrix calculation and softmax layer to perform convolution operation to get the final Note the matrix of space, following the same strategy as the channel attention module. The spatial attention module is depicted schematically in Figure 5.

**Figure 6:**
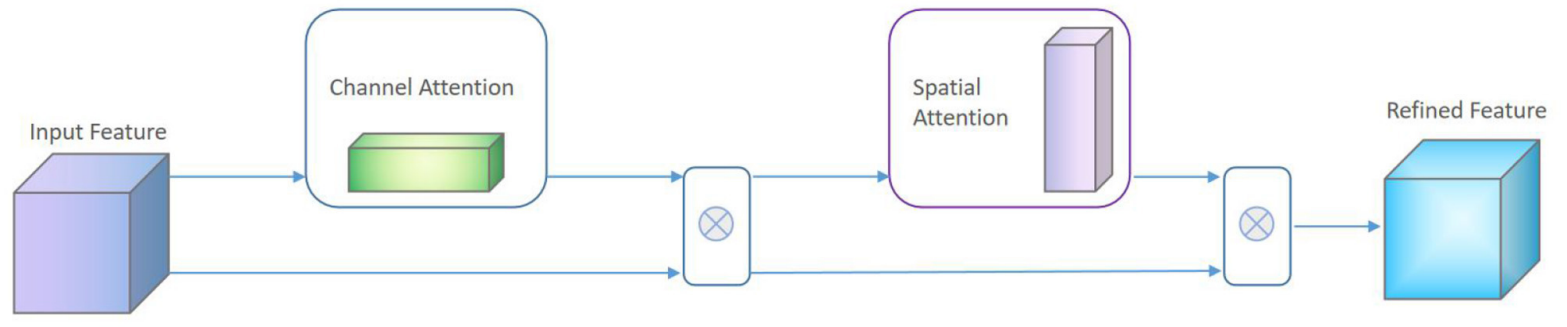
Schematic diagram of attention module.

Given a channel refinement feature $F_{C}\in R^{C\times H\times W}$, first pass the spatial attention module, use GAP and max-pooling to compress the feature map along the channel axis in parallel, and then obtain two feature vectors $F_{a}^{s}$ and $F_{m}^{s}$ with a dimension of 1 × *H* × *W*. Then the channel cascade is performed to merge the aggregation characteristics of the $F_{s}^{s}$. After the channel cascade, the convolutional layer with the kernel size of 3×3 is performed first, and then the PReLU and BatchNorm operations are performed to obtain the intermediate feature map $F_{p}^{s}$. During the convolution process, the step size is set to 1, and the filling value is also 1. In order to ensure that the size of the feature map remains unchanged, then:


(6) $$\begin{array}{l} F_{p}^{s}=Conv\left(F_{a}^{s};F_{m}^{s}\right)=\varphi^{3\times 3}\left(F_{a}^{s};F_{m}^{s}\right) \end{array}$$

where φ represents the convolution operation. Transform and transpose the middle $F_{p}^{s}$ to obtain two feature maps of *HW* × 1 and 1×*HW*, and then perform matrix multiplication and softmax operations to obtain the spatial attention matrix *A*_*s*_, and perform the softmax operation on each row of the spatial matrix, then:


(7) $$\begin{array}{l} A_{c}=soft\;\text{max}(F_{p}^{s}⊗{\left(F_{p}^{s}\right)}^{T}) \end{array}$$

where ⊗ represents matrix multiplication, and the following equation can be obtained:


(8) $$\begin{array}{l} A_{s_{i,j}}=\frac{\text{exp}\left({\left(F_{p}^{s}\right)}_{i}{\left(F_{p}^{s}\right)}_{j}^{T}\right)}{\sum_{\text{i}=1}^{HW}\text{exp}\left({\left(F_{p}^{s}\right)}_{i}{\left(F_{p}^{s}\right)}_{j}^{T}\right)} \end{array}$$

where *A*_*s*_*i,j*__ represents the influence of the *i*-th channel on the *j*-th channel. Finally, the channel refined *F*_*C*_ is multiplied by the channel attention matrix *A*_*s*_, and then the refined feature $F_{S}\in R^{C\times H\times W}$ is obtained through learning.


(9) $$\begin{array}{l} F_{S}=F_{C}⊕\left(\beta \left(A_{s}⊗F_{C}\right)\right) \end{array}$$

where β is a learnable parameter, and generally the initial value is set to 0 to reduce the difficulty of the convergence process of the first few training cycles. The spatial attention matrix *A*_*s*_ can be regarded as a position mask to focus on describing the most important part of the facial feature map. Therefore, the final structure of the attention module in the proposed algorithm is shown in Figure 6.

#### Interaction Mechanism

**Figure 7:**
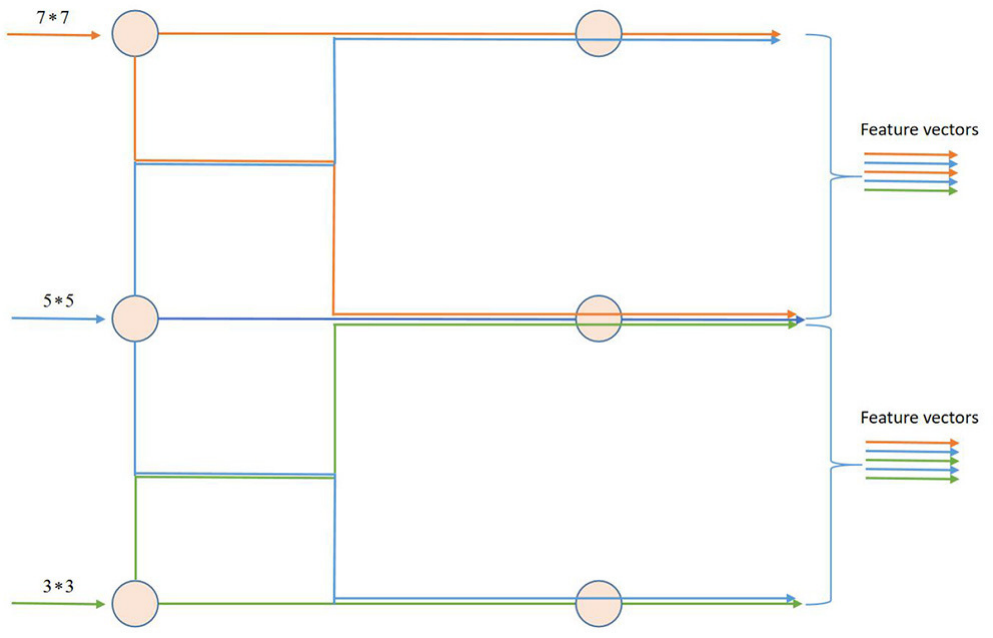
Schematic diagram of multi-layer feature interaction and feature flow.

Figure 7 depicts a schematic representation of multi-layer feature interaction and flow. The orange features are retrieved by the 7^*^7 convolution kernel, the blue features are extracted by the 5^*^5 convolution kernel, and the green features are extracted by the 3^*^3 convolution kernel. The multi-layer feature interaction module can capture the feature information between layers of different scales, and through the spatial attention mechanism, it can also extract the feature relationships between layers of different scales.

### Angular Distance Loss

**Figure 8:**
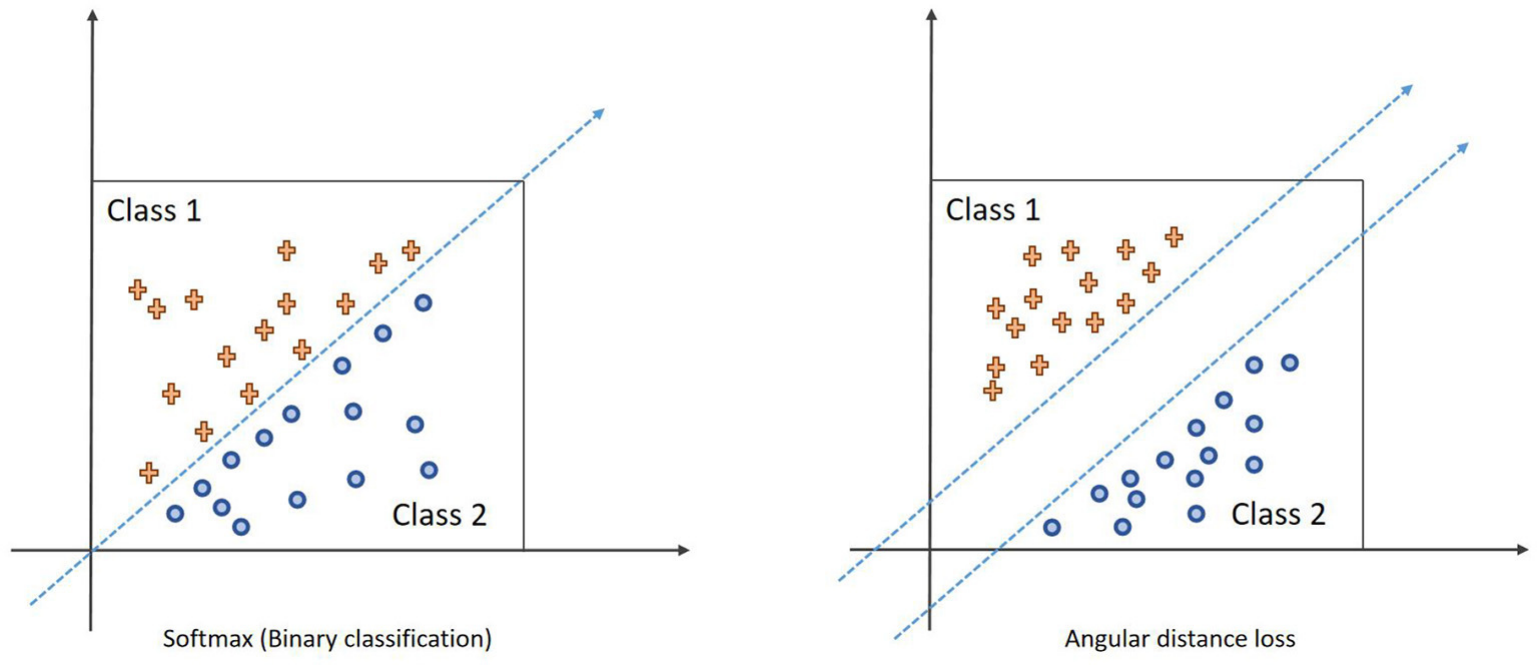
Schematic diagram of angular distance loss.

In order to further deal with the problems of large intra-class differences and high similarity between classes in facial emotion image recognition, we use the angular distance loss function to improve the capabilities of the proposed algorithm for feature separation between classes and clustering of features within classes. The calculation equation of angular distance loss is as follows:


(10) $$\begin{array}{l} L_{\text{ads}}=\text{-}\frac{1}{\text{m}}\overset{\text{m}}{\underset{\text{i}=\text{1}}{\sum }}\text{log}\frac{\text{exp}\left[\text{s}\times \text{cos}\left(\theta_{{\text{y}}_{\text{i}}}+\text{m}\right)\;\right]\;}{\text{exp}\left[\text{s}\times \text{cos}\left(\theta_{{\text{y}}_{\text{i}}}+\text{m}\right)\right]+\overset{\text{n}}{\underset{\text{j}=1}{\sum }}\text{exp}\left(\text{s}\times \text{cos}\theta_{\text{j}}\right)} \end{array}$$

where *s* is the scaling factor, cos(θ_y_i__ + m) is the angular distance, and m determines the size of the distance. The decision boundary of the softmax and the angular distance loss function in the case of two classifications is shown in Figure 8. The blue dashed line represents the classification decision boundary. Softmax classifies by angle, and the angle distance loss directly controls the distance of the classification decision boundary in the angle space through the decision margin m, thereby increasing the distance between classes, which is conducive to classification decision.

## Experiments and Results

### Experimental Setup

All experiments in this section are run on the same server to ensure a fair evaluation of the proposed algorithm. The server's specific configuration is as follows: the operating system is Windows 10, the GPU is NVIDIA GTX1080 (11G), the memory is 16G, and the CPU is AMD Ryzen 7 1700X; the deep learning development framework is Keras 2.1.5, install CUDA9+cudnn7, and the programming language is Python 3.6.5, Adam is the optimizer, and batch size = 16, learning rate = 0.001, Epochs = 300.

### Experimental Data Set

The FER2013 data set is the official data set for Kaggle's facial expression recognition competition in 2013. Because the majority of the images are downloaded from web crawlers, they will be compared because they comprise images of various ages, angles, and partially obscured images, among other things. There will be some inaccuracies, but it will be near to natural facial emotions. There are 35,887 photos in all in FER2013. The training set, public test set, and private test set are the three elements of the data set. The training set contains 28,709 photographs. Both the public and private test sets contain 3,589 images. Table 1 shows the data distribution in this data set, with the tags corresponding to the seven expressions numbered 0–6. Because the majority of the FER2013 data set comes from web crawlers, the background is more complex, making identification difficult.

**Table 1:** Data distribution of FER2013 dataset.

	**Angry**	**Fear**	**Disgust**	**Happy**	**Sad**	**Surprise**	**Neutral**	**Total**
Label	0	1	2	3	4	5	6	–
Train set	3,995	4,097	436	7,215	4,830	3,171	4,965	28,709
Validation set	467	496	56	895	653	415	607	3,589
Test set	491	528	55	879	594	416	626	3,589
Total	4,953	5,121	547	8,989	6,077	4,002	6,198	35,887

### Evaluation Method

Facial emotion recognition research mainly uses accuracy and confusion matrix as the evaluation indicators of the model. The accuracy rate represents the ratio of the number of correctly identified samples to the total number of samples, which can reveal the overall recognition ability of the model. The calculation equation is as follows:


(11) $$\begin{array}{l} Accuracy=\frac{\overset{C}{\underset{i=1}{\sum }}TP^{i}}{N} \end{array}$$

where *TP*^*i*^ represents the number of correctly classified samples in the *i*-th category, *C* represents the number of categories, and *N* represents the total number of samples.

The confusion matrix is a square matrix of size (*Z, Z*), in which the true label provided by the element *CP*_*ij*_ in the *i*-th row and *j*-column is the probability of the *i*-th category and the predicted label is the *j*-th category. The calculation equation is as follows:


(12) $$\begin{array}{l} CP_{ij}=\frac{n^{ij}}{n^{i}} \end{array}$$

where *n*^*ij*^ represents the true label is the *i*-th class and the predicted label is the number of samples in the *j*-th class, and *n*^*i*^ represents the total number of samples in the *i*-th class. By analyzing the confusion matrix, the accuracy performance of the model in each category can be measured.

### Experimental Results

We compared several well-known methods on the FER2013 data set to better evaluate the effectiveness of the proposed algorithm, and the results are shown in Table 2. Turan et al. (2018) proposed Soft Locality Preserving Map, a new and more effective manifold learning method that aims to control the diffusion level of different classes, effectively reducing the dimensionality of feature vectors and enhancing the extracted features. The improvement effect is not ideal for facial expression recognition distinguishing ability. Yang et al. (2018) proposed a facial expression recognition method based on residual expressions. Residual error learning is used to generate the residuals of the middle layer of the model. The residuals contain the expression components of any generated model of the input expression image, but the feature connection between the levels is not captured, and the classification effect is not good. The expression recognition rate of Shao and Qian (2019) in the two data sets is not high, and there is a problem that the recognition rate is low due to insufficient expression feature extraction. In addition, we also compared with InceptionV4, DNNRL, Multi-scale CNN, and Hybrid CNN-SIFT aggregator.

**Table 2:** Comparison of recognition rates of different algorithms on FER2013 dataset.

**Methods**	**Acc**
InceptionV4 (Szegedy et al., 2017)	0.7080
DNNRL (Kim et al., 2016)	0.7082
Multi-scale CNN (Wang and Yuan, 2016)	0.7282
SLPM (Turan et al., 2018)	0.7091
DeRL (Yang et al., 2018)	0.7264
Shao (Shao and Qian, 2019)	0.7114
Hybrid CNN-SIFT aggregator (Connie et al., 2017)	0.7340
**Ours**	**0.7416**

**Figure 9:**
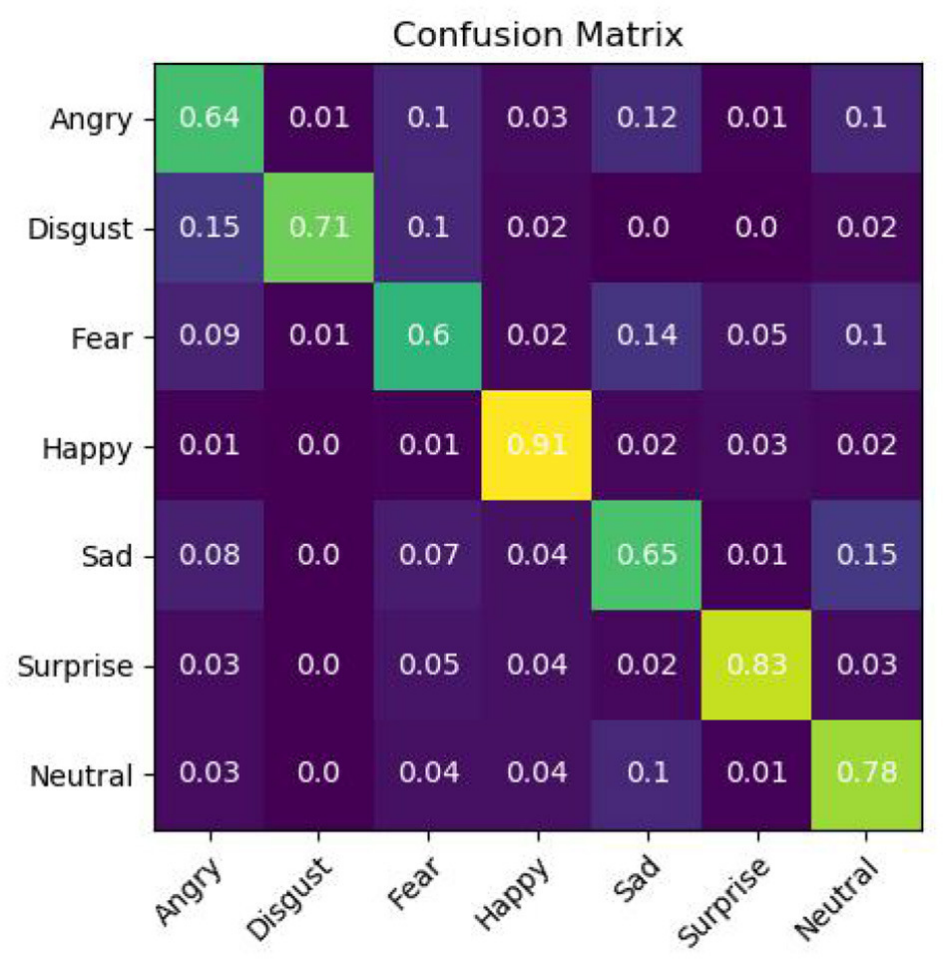
Confusion matrix of MIFAD-Net on FER2013 testing set.

**Figure 10:**
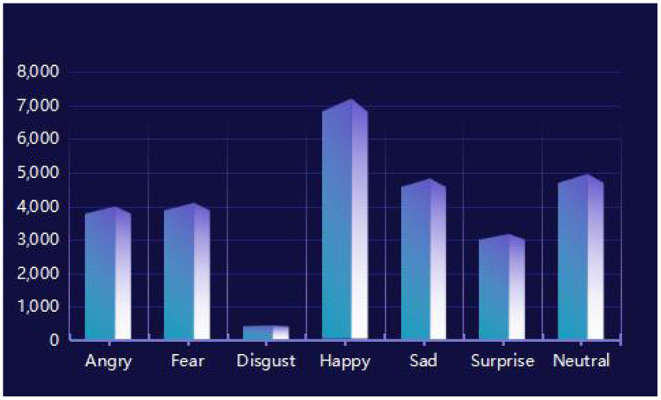
FER2013 training data distribution.

It can be seen from Table 2 that the proposed algorithm has achieved the best classification effect on the FER2013 data set in a complex environment. This is because the method in this paper makes full use of the multi-scale features of the multi-layer interactive feature fusion network, and integrates the cross-layer deep feature representation, captures the subtle changes in the deep level of expression, and restores the expression image gradually through multi-layer feature fusion. The useful feature information lost in the layer transfer process solves the problem of interaction between model layers and multi-layer feature fusion, and improves the network's ability to distinguish facial expressions caused by subtle changes in the corners of the mouth, eyebrows, and eyes. In addition, we propose Using the angular distance loss function effectively alleviates the problems of large intra-class differences and high inter-class similarity in facial expression recognition, and is more suitable for subtle facial expression classification. We also show the confusion matrix of the proposed algorithm on the test set in Figure 9. In addition, as shown in Figure 10, although the training data is unbalanced, the proposed algorithm overcomes this problem and achieves a competitive classification performance.

### Ablation Experiment for Different Loss Functions

To further verify the effectiveness of the angular distance loss function in the proposed algorithm, ablation experiments are set up in this section. We introduce Island loss and center loss for ablation studies. In addition, for fair comparison, all experiments are performed in the same environment. The results of the ablation experiment are shown in Table 3.

**Table 3:** Results of ablation experiments for different loss functions on FER2013 dataset.

**Methods**	**Acc**
Softmax	0.7211
Island+Softmax	0.7385
Center loss+Softmax	0.7294
**Ours**	**0.7416**

It can be seen from Table 3 that the best classification performance is obtained by using the angular distance loss. In addition, we find that the Island loss is better than the center loss, and the softmax performance alone is the worst. Because Softmax predicts the probability of each category, it does not optimize the distance between the classes and the class, which leads to the lack of distinction between features. In order to reduce the difference in features within the class, the authors (Cai et al., 2018) proposed the optimization and improvement of Center and Island Loss. Island Loss increases the constraints of facial expression features to make the distance between classes larger, thereby improving the classification performance.

### Ablation Experiment for Multi-Layer and Multi-Scale

A parallel three-branch network is used in the proposed algorithm. An ablation experiment is set up in this section to further prove its effectiveness. For comparison, we add two-branch and four-branch networks. The ablation experiment's results are shown in Table 4.

**Table 4:** Results of ablation experiments for multi-layer and multi-scale on FER2013 dataset.

**Methods**	**Acc**
Two-branch	0.7105
Four-branch	0.7391
**Ours**	**0.7416**

Table 4 shows that using a two-branch network reduces the model's classification performance significantly, whereas using a four-branch network does not improve classification performance. As a result, the proposed algorithm is proven to be effective.

### Ablation Experiment for Attention Mechanism

This section sets up an ablation experiment to test the effect of the proposed algorithm's attention mechanism on classification performance in order to verify its effectiveness. The term “No-attention” refers to the lack of use of the attention mechanism. Table 5 shows the results of the ablation experiment.

**Table 5:** Results of ablation experiments for attention mechanism on FER2013 dataset.

**Methods**	**Acc**
No-attention	0.7262
**Ours**	**0.7416**

It can be seen from Table 5 that if the attention mechanism is not used, the classification performance of the model will be reduced by 1.97%. Because facial expressions consist of muscle movements in specific parts of the face. The features produced by these local areas contain the information that best describes expressions. Therefore, using the attention mechanism to quantify the importance of each spatial position in the feature map and focusing on the areas with rich emotional information is beneficial to the recognition task.

### Ablation Experiment for Feature Fusion Strategy

To further verify the influence of the feature fusion strategy on the experimental results, an ablation experiment was carried out in this section. “Add” stands for addition strategy, “C” stands for concat strategy, and “Mul” stands for multiplication strategy. The results of the ablation experiment are shown in Table 6.

**Table 6:** Results of ablation experiments for feature fusion strategy on FER2013 dataset.

**Methods**	**Acc**
Add	0.7325
Mul	0.7298
**Ours (C)**	**0.7416**

It can be clearly seen from Table 6 that the Concat strategy used by the proposed algorithm achieves the best results. Secondly, the addition and multiplication is better than the multiplication strategy, which proves that the extraction of multi-scale features effectively improves the classification performance of the proposed algorithm, and also further prove the superiority of the proposed algorithm.

## Conclusion

In this paper, we propose a novel multi-layer interactive feature fusion network model with angular distance loss. First, a multi-layer and multi-scale module is designed to extract the global and local features of facial expressions to capture part of the feature relationships between different scales, thereby enhancing the model's ability to discriminate subtle features of facial expressions. Secondly, in view of the problem of loss of useful feature information due to layer-by-layer convolution and pooling of convolutional neural networks, a hierarchical interactive feature fusion module is designed. The attention mechanism is used between convolutional layers at different levels to control the network. Strengthen the saliency information of different characteristics in the Internet and suppress irrelevant information, thereby improving the discriminative ability of the network. Finally, for the problem of large intra-class differences and high similarity between classes in facial expression recognition, we use the angular distance loss function to improve the capabilities of the proposed algorithm for feature separation between classes and clustering of features within classes. We conducted comparison and ablation experiments on the FER2013 data set. The results illustrate that the proposed MIFAD-Net outperforms a number of well-known methods and is highly competitive.

## Data Availability Statement

Publicly available datasets were analyzed in this study. This data can be found here: https://www.kaggle.com/c/challenges-in-representation-learning-facial-expression-recognition-challenge/data/.

## Author Contributions

WC: conceptualization, methodology, software, and writing. JM: investigation. MG: data curation, software, and validation. RL: data curation and investigation. All authors contributed to the article and approved the submitted version.

## Funding

This research was funded by the Scientific Research Program of Education Department of Hubei Province, China (D20184101), Higher Education Reform Project of Hubei Province, China (201707), East Lake Scholar of Wuhan Sport University Fund, China and Hubei Provincial University Specialty subject group construction Special fund, China.

## Conflict of Interest

The authors declare that the research was conducted in the absence of any commercial or financial relationships that could be construed as a potential conflict of interest.

## Publisher's Note

All claims expressed in this article are solely those of the authors and do not necessarily represent those of their affiliated organizations, or those of the publisher, the editors and the reviewers. Any product that may be evaluated in this article, or claim that may be made by its manufacturer, is not guaranteed or endorsed by the publisher.
